# Physical activity and diabetes in german primary care: a qualitative interview study with individuals living with diabetes

**DOI:** 10.1186/s12875-026-03344-z

**Published:** 2026-04-29

**Authors:** Nicole Lindner, Louisa Friedrich, Nele Kornder, Annette Becker, Veronika van der Wardt

**Affiliations:** Department of Primary Care, University of Marburg, Karl-von-Frisch-Str. 4, Marburg, 35043 Germany

**Keywords:** Primary Health Care, Diabetes Mellitus, Type 2, Exercise, Locus of Control, Patient Compliance

## Abstract

**Background:**

Physical activity (PA) plays a crucial role in the management of Type 2 Diabetes Mellitus (T2DM). Despite its known benefits, many individuals living with T2DM face challenges in maintaining regular PA. General practitioners (GPs) are often seen as key facilitators of lifestyle change, yet little is known about how individuals living with T2DM perceive PA and the role of GPs in promoting it. This study aims to explore the personal perceptions, motivators, and barriers related to PA among individuals living with T2DM, and to understand their expectations for GP support within the primary care setting.

**Methods:**

A qualitative study was conducted in primary care settings in Germany. Participants were recruited from self-help groups and GP practices across Hesse, Bavaria, and Berlin. Semi-structured interviews were conducted with 13 individuals living with T2DM. Interviews were transcribed verbatim and analysed using an inductive-deductive thematic analysis approach (Braun and Clarke) informed by the Health Belief Model.

**Results:**

Participants recognised PA as essential for diabetes management and reported diverse experiences and challenges. Motivators included fear of complications, self-efficacy, enjoyment, goal-setting, and perceived health benefits. However, self-efficacy played a complex role—while it encouraged PA, it also increased feelings of personal responsibility, sometimes resulting in stress, guilt, or excessive self-monitoring. Barriers included comorbidities, lack of companionship, time constraints, and reluctance. Participants valued GP support, valuing a partnership-relationship, while also feeling motivated through authorities. Suggestions for GP involvement included tailored PA counselling, regular follow-up, structured goal-setting, and addressing emotional aspects of diabetes.

**Conclusion:**

PA was widely acknowledged as important, yet its adoption was influenced by personal and contextual factors. Self-efficacy played a dual role, acting as both a motivator and a psychological burden. GPs were seen as important in PA promotion, but their support needed to be flexible and personalised.

**Supplementary Information:**

The online version contains supplementary material available at 10.1186/s12875-026-03344-z.

## Background

Diabetes is one of the fastest growing health-emergencies of the 21st century, with prevalence rates rising sharply over the past decades. In 2021, an estimated 10.5% of the global adult population was living with diabetes, with the highest rates observed in high-income countries. Type 2 Diabetes Mellitus (T2DM) is the most common form of diabetes, accounting for over 90% of all cases worldwide [1]. Similarly, in Germany the prevalence of diabetes was 8.9% among adults in 2020, with rates increasing considerably with age—by 65 years, one in five individuals had a known diagnosis. The prevalence has risen markedly from 5.2% in the late 1990s to current levels.

Diabetes mellitus is a group of chronic metabolic diseases characterised by persistent hyperglycaemia resulting from defects in insulin secretion, insulin action, or both. In T2DM, hyperglycaemia typically arises from insulin resistance in target tissues combined with progressive pancreatic β-cell dysfunction. Chronic hyperglycaemia is associated with substantial long-term organ damage and a wide spectrum of complications, classically divided into microvascular complications (retinopathy, nephropathy, and neuropathy) and macrovascular complications (coronary heart disease, cerebrovascular disease, and peripheral arterial disease). Cardiovascular disease is the leading cause of morbidity and mortality among individuals living with T2DM. Beyond these complications, there is increasing recognition of additional diabetes-associated conditions, including cognitive decline, metabolic dysfunction–associated liver disease, elevated risk of several cancers, and—especially in older adults—frailty and functional impairment. Optimising glycaemic management to maintain near-normal glucose levels can prevent or delay the development of complications [1–3].

Approximately 91.3% of individuals living with T2DM in Germany receive care through general practitioners (GPs), emphasizing the central role of primary care in diabetes management and intervention [4]. The treatment situation is complex: GPs are under pressure to achieve specific surrogate outcomes – such as HbA1C levels – in their patients, yet often report a sense of resignation when these targets remain unattainable. Moreover, the perceived devaluation of individuals living with T2DM may reinforce entrenched power dynamics, potentially undermining collaborative care [5].

Physical activity (PA) is a cornerstone in the management of Type T2DM, offering benefits such as improved glycaemic control, weight management, and reduced cardiovascular risk [4, 6, 7]. Despite these well-documented benefits, a substantial proportion of individuals living with T2DM do not engage in the recommended levels of PA. Approximately 70% of adults with T2DM fail to achieve the minimum of 150 min of weekly PA recommended for self-management [8, 9].

Qualitative research indicates that counselling on PA in diabetes is shaped by multiple interacting factors across health systems and settings. For example, an ethnographic study from Denmark found a mismatch between clinicians’ predominantly health- and risk-focused framing of PA and participants’ everyday priorities (e.g., enjoyment, pain, family obligations), which can complicate engagement with PA recommendations [10]. Studies from other European primary-care contexts similarly describe PA counselling as challenging, highlighting tensions around responsibility and motivation and the difficulty of translating guideline recommendations into patients’ everyday lives (e.g. neighbourhood characteristics, health-related problems, and insufficient social support) [11–13]. In German primary care, qualitative research in other chronic conditions (e.g., chronic back pain) suggests that physical-activity counselling is hindered by limited time, scarce local opportunities, comorbidities, and patients’ negative emotions such as anxiety or frustration. Counselling and the doctor–patient relationship also appear to influence each other [14]. The evidence regarding the perspectives of individual individuals living with diabetes is limited.

Therefore, the aim of our study was to explore the personal perceptions, ideas, and wishes of individuals living with T2DM regarding PA in primary care settings. Specifically, we sought to understand what type of support and lifestyle counselling of individuals living with T2DM desire from their GPs to enhance PA.

## Methods

This manuscript is conducted in accordance with the consolidated criteria for reporting qualitative research (COREQ) [15].

### Study design

We conducted a semi-structured interview study with individuals living with T2DM. The researchers chose this semi-structured interview approach to openly explore the wishes, expectations, and personal views of individuals living with diabetes. Following an explanation of the nature and possible consequences of the study, informed written consent was obtained from all participants. The protocol followed the tenets of the Declaration of Helsinki and we obtained ethical approval from the Ethic Boards of the department of Medicine of the University of Marburg (“Ethikkommission Marburg”) (AZ142/20) [16]. The research team is multidisciplinary (with backgrounds in medicine and psychology). LF, a female medical student, who undertook this study for her doctoral degree. NL, NK, AB and VvdW are female researchers experienced in qualitative research, NL, NK and AB are also practising GPs. LF received training in qualitative methods during regular meetings of the working group, where she presented her project and obtained feedback from all members. She conducted two pilot interviews to refine the interview guide. Regular feedback sessions on interview style and reflexivity were carried out with VvdW, and during the analysis LF received additional guidance from VvdW, NL and NK. All researchers reflected on their personal experience of PA and, where relevant, their experience of working as a GP. LF knew a few participants, as they were recruited in her hometown where her father had previously worked as a GP. To manage this potential influence, we applied several strategies: LF and the research team discussed possible effects of familiarity on the interview process; LF conducted all interviews in a structured and consistent manner using the interview guide; and the analysis was carried out collaboratively to ensure that emerging themes were not shaped by prior acquaintance with individual participants. The study process underwent an internal peer review by the entire working group and an external peer review through a presentation at the German GP conference. A patient advisory board supported the project in particular in analysing the themes.

### Study sample

We employed a purposive sampling method to ensure the inclusion of diverse viewpoints. The inclusion criteria were diagnosis of T2DM, a minimum age of 18 years, sufficient proficiency in German to understand the study information, provide informed consent, and participate in an interview without an interpreter. No additional exclusion criteria were applied. We recruited via self-help groups and GP surgeries in the states of Hesse, Bavaria and Berlin (Germany), using flyers and email. For self-help groups, the invitation email was forwarded by the group coordinators via their internal mailing lists; we did not collect email addresses ourselves. A prior relationship existed with a few participants, as LF personally knew some individuals recruited via her father’s former GP practice in her hometown. Potential participants contacted the research group via mail/telephone and were provided with written and verbal information on the study. Recruitment was terminated when thematic saturation was reached, meaning that no substantially new codes or themes emerged from the data. This judgment was made through ongoing analysis during the interview process. The decision to stop recruitment was discussed and agreed upon within the research team (LF, VvdW). No interviews were discontinued. One individual initially agreed to participate but did not respond to follow-up scheduling requests, and therefore no interview took place.

### Data collection

We developed an interview-guide tailored to our research questions. Initially, we undertook two pilot interviews with individuals living with T2DM to refine the interview-guide. After the first three interviews, we further adjusted the interview guide to enhance clarity and add more insightful questions. An overview of main topics and corresponding sample questions are presented in Table 1. The complete interview-guide can be found in supplement 1. Demographic data were collected using a paper-based questionnaire. Interviews were conducted by LF between October 2020 and February 2021. Due to COVID-19 restrictions, all interviews were carried out via telephone. No repeat interviews were conducted. In one interview, the participant’s spouse was present in the background during the conversation. Interviews and analyses were completed in German as all participants could speak and understand German. Transcripts were not returned to participants for comment, correction or feedback in order to minimise participant burden and to preserve participants’ spontaneous perspectives.

**Table 1:** Interview guide. The original interview guide was German and was translated into English for publication. PA: physical activity

Main topic	Sample questions
PA status	How satisfied are you with your physical activity in everyday life? What do you aim to achieve through physical activity?
Diabetes and PA	What impact does diabetes have on your physical activity? How physically active were you before your diagnosis? Do you feel that you can positively influence your condition? How?
PA and primary care	Do you think the general practice is an appropriate setting to provide advice on promoting physical activity? To what extent does the relationship with the GP play a role in lifestyle counselling? Where could the GP start to motivate you to move more? Where is your “sensitive spot”? How could the GP reach you and draw out more motivation for movement? Which advice from your GP has already helped you to be more physically active
Ideas to promote PA	Depending on what the interview partner mentioned discussion on different ideas: - Primary care: What should an offer in primary care ideally look like? - Information material: Which type of information would be good? - Classes: What do you want to learn about? - Financial support for PA: Under what circumstances would you accept and use it? Take a moment again. Do you have any ideas on how your GP could support you?

### Data analysis

Interviews were audio recorded and transcribed verbatim. Quotes were translated for publication into English by NL with support of chatGPT-4 (OpenAI). All translations were subsequently double-checked by NL and reviewed within the research team. Additionally, field notes were taken. Data were anonymised and all participants received pseudonyms (fictitious names according to the recommendation on pseudonymisation) [17]. Data were managed in MAXQDA (2020 and 2022) and coded by consensus. Following the reflexive thematic analysis method by Braun and Clarke data was analysed by LF and NL using a deductive– inductive approach; with interview questions supporting theme development (deductive) but participant answers allowing new themes to emerge (inductive) [18, 19]. We selected Braun and Clarke’s method for its flexibility to adapt to emerging study aspects and the researchers’ comprehensive experience with the approach. Interviews were analysed using the six-step thematic approach, including familiarization, coding, theme development and refinement, and synthesis of findings. LF conducted the initial coding of all transcripts, and these codes and preliminary themes were discussed with VvdW. NL then reviewed the coding and themes, recoded the data, and developed overarching themes. The whole research team subsequently discussed and refined the themes to ensure a rich and reflexive interpretation of the data. While we employed reflexive thematic analysis to allow themes to emerge inductively, our interpretation was informed by constructs from the Health Belief Model [20]. This combined approach aligns with reflexive thematic analysis, which acknowledges the researcher’s active role and values flexibility in the generation of themes.

To enhance credibility, coding and theme development were discussed within the study team. Interpretations were further reviewed with input from a patient advisory board. Transferability was supported by providing detailed descriptions of participants, context, and setting. Dependability was ensured through systematic coding procedures, the use of field notes, and documentation of analytic decisions throughout the process.

## Results

We conducted interviews with 13 individuals living with T2DM. The duration of the interviews ranged from 26 to 66 min. The participants included seven women and six men, aged between 38 and 80 years. PA levels varied widely among participants, from minimal household activities to regular strength training and walking 15 km per day. Further details on sociodemographic data can be found in Table 2.

**Table 2:** Overview of sociodemographic participants. *Hildegard and Heinrich were married. PA: Physical activity

Pseudonym	Age	Gender	School-leaving certificate	Age at diagnosis (years since diagnosis)	Self-reported PA
Ludwig	70–89	man	Intermediate school	41 (34)	Nordic walking in a group several times a week
Heidi	70–89	woman	Intermediate school	61 (12)	Gymnastics & running in groups (1x/week)
Gerhard	70–89	man	Lower secondary school	58 (17)	Stepper (1x/week) and gardening
Lotte	60–79	woman	Lower secondary school	67 (1)	PA during housework
Matthias	60–79	man	Intermediate school	55 (6)	Housework and gardening
Hildegard*	70–89	woman	Lower secondary school	66 (14)	Short daily walk (approx. 1 km)
Heinrich*	70–89	man	Lower secondary school	69 (11)	Short daily walk (approx. 1 km)
Martina	50–69	woman	Lower secondary school	48 (3)	PA during housework & walk at work
Karin	70–89	woman	Elementary school	65 (9)	Short walks several times a week
Sven	30–49	man	Intermediate school	39 (3)	Club sports several times a week
Melanie	30–49	woman	German A-levels	20 (18)	Even housework is difficult
Beate	30–49	woman	Lower secondary school	41 (1)	Daily walk & walk at work
Theo	30–49	man	Lower secondary school	31 (9)	Strength training (3x/week) & lots of walk at work

Interview partners reflected on the following themes:

1. Influence of diabetes on PA,
2. Motivators of PA,
3. Pressure and Self-Efficacy (Self-Efficacy Paradox),
4. Barriers of PA,
5. Facilitators of PA,
6. Role of GP.

**Fig. 1:**
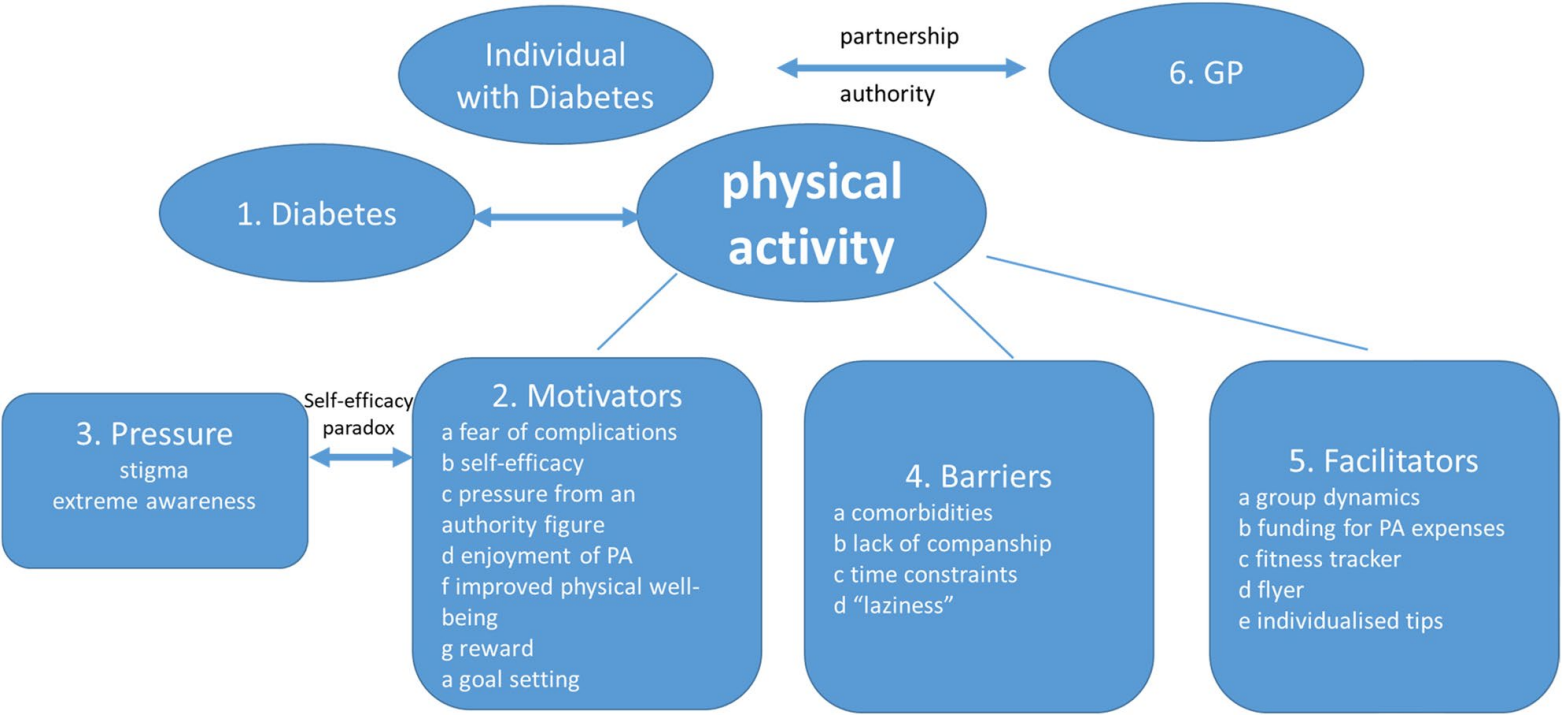
Thematic map illustrating themes and corresponding subthemes

Please see Fig. 1 for a thematic map of themes and subthemes.

In the following, we want to present the main aspects of our analysis.

### Influence of PA on diabetes and vice versa

Generally, interview participants identified PA as one of the key components of diabetes management.

“But movement is crucial for managing blood sugar. You can do what you want; I didn’t quite believe it at first, but it’s true.” (Heinrich, 70–89 years of age).

However, opinions varied on how the diagnosis of diabetes influences PA. For some, a diagnosis of diabetes marked a turning point that led to an increase in their PA.

“It has increased. I run even more now.” (Heidi, 70–89 years of age).

While others noted that they had been active throughout their lives.

“I have actually been doing competitive sports all my life.” (Sven, 30–49 years of age).

Particularly older individuals living with diabetes expressed that they do not plan to alter their lifestyles due to the diagnosis, feeling content with how things are.

“I don’t think I will change anything now. I am as I am, not unstable but satisfied.” (Hildegard, 70–89 years of age).

Rather than focusing on lifestyle changes, those participants emphasised what mattered most to them, such as being with loved ones.

### Motivators of PA

Participants provided comprehensive insights into the factors that actually or potentially motivate them to engage in PA. Motivators, as defined in this study, refer to internal or external motives that prompt individuals to engage in PA.

A primary motivator for individuals living with diabetes appeared to be fear of complications(2a). This subtheme emerged strongly, with participants expressing profound anxiety about the potential severity of their condition, including fears of death.

“Well, if I’m being honest, it’s really the fear of the consequences of the disease, right? So there, when you hear about everything that can happen to your body if you don’t take care of it. That’s what really motivates me to do something about it, you know?” (Lotte, 60–79 years of age).

Additionally, interview partners described a feeling of self-efficacy as motivator (2b). They shared feelings of empowerment when they managed their health proactively.

“Oh my goodness, then I went home and looked in the mirror and said, ‘Well done!’ (laughs). That was my reward.” (Heid, 70–89 years of age).

While pressure from an authority figure (2c) was identified as motivating, it did not appear to play a notable role in the doctor-patient-relationship.

“Yes, well, if he [GP] responds a little bit like this (…) saying, ‘Now just get started’ or ‘Now run and do it,’ or something like that. I think that helps, when the doctor says that to you.” (Lotte, 60–79 of age).

Another significant incentive discussed was goal setting (2d) across various dimensions. Participants set goals not only regarding the quantity of PA they aimed to achieve but also medical goals such as reducing or even eliminating the need for medication, and physical goals like weight loss. One participant described a combination of physical and medical goals:

“I’m automatically trying to reduce my weight (…), I’m also trying to reduce the amount of insulin I inject today as a base, perhaps just a tiny bit.” (Ludwig, 70–89 years of age).

Participants also reported that improved physical well-being (2f) motivated them to maintain or increase their activity levels. Views on rewards (2 g) as a motivator for physical activity were mixed, indicating that while some found rewards encouraging, others completely rejected the idea.

“What I treat myself to is a nice wheat beer, non-alcoholic, I should add. That’s the best thing. And a nice shower after sport and then drinking a non-alcoholic beer, a wheat beer.” (Gerhard, 70–89 years of age).

An overview of motivators and illustrating quotes can be found in in Table 3.

**Table 3:** Subthemes for motivators for PA

Motivator	Illustrative quote
2a fear of complication	“I really do it because of my disease, namely arterial disease [complication of diabetes], because otherwise I think I would have passed away much earlier. So I try to fight against it and the partial clogging that are there. (…) It could happen, but then it’s my fate. It’s this cursed disease that can strike anytime, any hour because the arteries are calcified. I’ve seen the chunks of calcium in the arteries on a full-body scan, and if a chunk breaks loose, I’m gone instantly.” (Ludwig, 70–89 years of age)
2b self-efficacy	“It’s not always possible, there are periods when you let things slide, clearly, but you have to take responsibility for that, I tell you, and you can’t pass it off to anyone else.” (Ludwig, 70–89 years of age)
2c pressure from an authority figure	“He [GP] just has to threaten me with injections. If he says, ‘Watch out, if it doesn’t get better, we’ll have to start with injections because it’s too high.’ That would be the only motivation for me to maybe move more.” (Matthias, 60–79 years of age)
2d enjoyment of PA	“It’s so much fun, you feel totally different than when you’re just sitting on your butt all the time, it’s really nothing.” (Heinrich, 70–89 years of age)
2e goal setting: PA goals	Yes, that’s right. A specific goal for me is that I say, okay, after breakfast on Sunday, I go to the attic and ride a bike. 10 km, that’s what I’ve set for myself. And I stick to it and have been doing it for three weeks now. (Gerhard, 70–89 years of age)
2e goal setting: medical goals	“So that the values might get even better, that I no longer have to take pills. I have to take Metformin, those are the pills. Metformin 500 they’re called. My goal is to completely get rid of diabetes.” (Gerhard, 70–89 years of age)
2e goal setting: physical goals	“It was really about weight reduction and endurance.” (Theo, 30–49 years of age)
2f improved physical well-being	“Just noticing that my shoes were tight before I went into the water and after the half hour, it was only half an hour of aqua fitness, my shoes were loose. Just that half hour in the water moving properly without getting out of breath did my body so much good.” (Beate, 30–49 years of age)
2 g reward	“Right? Thinking: ‘Okay, if I allow myself a reward, like eating a piece of chocolate after I’ve had a good run,’ that really helps me, I think it does for everyone, right?” (Lotte, 60–79 years of age) “I actually reject such things. I never did that with my children either.” (Heidi, 70–89 years of age)

### Pressure and self-efficacy

While self-efficacy was described as a motivator for PA (please see 2b), it seemed to have also the potential to create pressures. Some individuals described self-imposed stress related to constant self-monitoring and performance tracking, reflecting a heightened sense of personal responsibility for their PA behaviour.

“For over 14 or 13 years, I have been constantly using (…) pulse watches in various forms, and I believe I have been wearing them around the clock on my wrist for about 5 or 6 years now. As a result, I always have a very good overview of my activities on my phone. (Ludwig, 70–89 years of age)

This intense monitoring illustrates what we term a self-efficacy paradox: high perceived competence and responsibility simultaneously acting as a source of motivation and pressure.

In one case, this sense of responsibility extended beyond the individual to others, particularly family members. One participant (Heidi, 70–89 years of age) described the feeling of responsibility of preventing diabetes in their children, which coincided with the development of an eating disorder of her daughter. However, she did not discuss the potential causes of the eating disorder.

“You try, especially when diabetes runs in the family, to pass on this caution to the next generation: ‘Listen, pay attention to this and that.’ (.) And my daughter, she developed anorexia when she was 12 or 13 years old.” (Heidi, 70–89 years of age).

Other interviewees highlighted how strong expectations of self-management were accompanied by experiences of blame and stigma. Despite striving to manage their diabetes, they felt blamed and stigmatised for perceived “failures” in self-management. One participant recounted being directly accused during her pregnancy.

“You have already caused your daughter to have a heart defect, please make sure you do not pass on diabetes to her as well.” (Beate, 30–49 years of age).

This sense of blame extended beyond diabetes management to broader social judgments about parenting and responsibility, as this participant reflected also on how quickly others attribute fault based on a child’s appearance.

“Because if your child is too think, people assume, ‘You’re eating all their food. You’re not giving them anything’. And if they’re too fat, people assume, ‘You’re juts feeding them Snickers’.”

### Barriers of PA

Interview participants identified several key barriers, including limiting comorbidities, lack of companionship, time constraints, and personal factors (e.g. perceived “laziness”), that hinder their engagement in PA.

Comorbidities (4a) were highlighted as a significant challenge, as some individuals expressed physical limitations that directly affect their ability to be active. For instance, on interviewee shared,

“Not too fast (…) otherwise, you can’t breathe (…) Or if I run too slowly, maybe, I don’t know if that’s related to my back hurting.” (Karin, 70–89 years of age).

The absence of companionship (4b) emerged as a key barrier, as participants described social support as central to sustaining motivation for PA. As one participant noted:

“But when I’m alone, I have to say, I don’t feel motivated.” (Lotte, 60–79 years of age).

Additionally, time constraints (4c) were mentioned as barrier for engaging in PA, particularly among those balancing demanding careers and family obligations.

“Exactly, due to time constraints, it wouldn’t be possible with my job and everything like that.” (Sven, 30–49 years of age).

Personal challenges such as inherent “laziness” (Karin, 70–89 years of age) (4d) were also discussed, with participants acknowledging the difficulty in mustering enthusiasm for PA.

“Right now - I can’t - I don’t know why, but right now I just can’t bring myself to do it. I just can’t muster the effort right now.” (Martina, 50–69 years of age).

### Facilitators of PA

Interviewees provided comprehensive insights into various facilitators that ease the initiation and maintenance of PA. Facilitators, as defined in this study, refer to specific conditions or supports that improve access to and engagement in PA.

One central part was that interview partners preferred engaging in PA in groups (5a), such as in organised classes or with friends. They found group settings motivating for various reasons, including the sense of commitment and the enjoyment of meeting others.

“I would definitely join again [walking group], because it’s more fun with two people than walking around alone.” (Gerhard, 70–89 years of age).

Opinions on other potential facilitators of PA were mixed. Some participants felt motivated by funding for PA related expenses, such as gym memberships or equipment like Nordic walking sticks, while others completely rejected this idea.

“No (loudly), I need them [walking shoes]—I can buy them myself, for heaven’s sake. Don’t give me that—I don’t want that. (…) I’ll do that myself. I don’t want to use my health insurance for that. For heaven’s sake. It all costs enough money as it is.” (Heinrich, 70–89 years of age).

Similarly, while some saw flyers as motivational tools— with one participant (Heidi, 70–89 years of age) even distributing them at doctors’ offices—others viewed them as “a waste of money” (Matthias, 60–79 years of age).

Additionally, one participant (Beate, 30–49 years of age) proposed a clear strategy for facilitating access to PA. She suggested initially assessing personal preferences and accessibility, and then providing tailored advice and resources accordingly.

An overview of facilitators can be found in in Table 4.

**Table 4:** facilitators to engage in PA

Facilitator	Illustrative quote
5a group dynamics	“First of all, it’s good for the soul when you get together with other people, right? It’s fun, you get to talk about something different. It’s actually nice. And that would motivate me much more than if someone says, ‘You have to do this and that at home for half an hour every day.’ I know for sure that I would never follow through with that. (Lotte, 60–79 years of age)
5b funding for PA expenses	“A financial aspect plays a role for many people (…). So definitely a lot more people would engage in sports if that were addressed.” (Sven, 30–49 years of age) “It’s in everyone’s interest to take care of their own health, and you can’t expect the general public to always pay a premium. Because that money would again come from the public—from somewhere, right? From tax revenues or health insurance contributions. “ (Matthias, 60–79 years of age)
5c fitness tracker	“Then I would probably be proud of myself when I say in the evening or when I look at the thing and think: ‘Oh, today you walked I don’t know how many steps, right? That might motivate me—for myself, you know?—to say: ‘Well, you were quite lazy today. Now get up and do something more, right?’ That might help me, and that’s why I’ve already thought about really buying one of those devices.” (Lotte, 60–79 years of age) “For me personally, I must say I don’t need such a device.” (Matthias, 60–79 years of age)
5d flyer	“If they were available at the doctor’s office (…) then you automatically take one [flyer] with every visit (…) that would be great. Everyone would grab one and take a look.” (Lotte, 60–79 years of age) “I think it’s a waste of money for me. (…) I get the brochures, I take a look, and then zap, they’re gone.” (Matthias, 60–79 years of age)
5e Individualised tips	“So, I can really imagine, if you have no idea yet, that you could find out what suits you through a quiz, and then you can also build a great marketing strategy around it by saying: ‘For you, aqua fitness at the swimming pool around the corner might be suitable, or there is a gym within a five-minute walk that offers classes that really match your fun profile. But if that doesn’t fit into your current life phase, here are five exercises that you can easily incorporate into your daily routine at home.’ Like doing jumping jacks while brushing your teeth or something like that. Things that you’ve probably heard of before.” (Beate, 30–49 years of age)

### Role of GP

Interview partners reflected on their relationship with their GP and how they could support PA. Generally, the interviewees described a good and understanding doctor-patient relationship. Through regular and sometimes long-term care, many individuals living with T2DM developed a trusting relationship with their GPs. Suggestions and advice from GPs were perceived by most individuals living with T2DM as credible and trustworthy.

“I am very satisfied with my GP in that regard. He’s good and knows how we do things; we practically do the blood work, then have a discussion, and look at the values, and then he says, ‘You should do something again.’ I say, ‘I know, if I could. If I didn’t have the whole situation [comorbidities, e.g. knee operation], I would do something.’ And he understands that, and as I said—the connection to the general practitioner because of the situation, to me, personally, is very good.” (Gerhard, 70–89 years of age).

Interestingly, even though some interview partners described pressure from an authority figure as motivating (see also 2c), they valued a partnership-based doctor-patient relationship. However, some interview partners expressed a desire for GPs to use their authority more assertively to highlight health urgencies and provide specific advice.

„It’s already very important for a general practitioner to point it out and say: ‘Look here, things are looking pretty bad for you, you need to change something.” (Beate, 30–49 years of age).

Interview partners articulated specific ideas how GPs could facilitate PA. As highlighted in Sect.  5 one interviewee (Beate, 30–49 years of age) detailed a structured approach for GPs, from assessing the current situation and need to provide personalised opportunities to increase PA. Others expressed further ideas, such as the development of an exercise plan to initiate PA. These agreements could provide a level of commitment.

“This would certainly help, right? At least in the short term. It would definitely motivate me at first because then I see: yes, he cares, he’s doing something, and you’re just standing there, that wouldn’t sit right with me, right? So, I would push myself more […], but (…) the duration, that is what I think the problem is.” (Matthias, 60–79 years of age).

While there were concerns that this might not have a long-term effect on exercise behaviour, the proposed solution was to maintain ongoing communication about the agreements made and to collaboratively address any difficulties that arise.

“Well, every six months, it would definitely be worthwhile for the GP to just check in: Has it worked? Could anything be implemented? What’s stopping it from working?” (Melanie, 30–49 years of age).

## Discussion

### Principal findings

This study offers deep insights into the personal perceptions, ideas, and wishes of individuals living with T2DM on PA within primary care settings. Interviewed individuals living with T2DM clearly recognised PA as a fundamental component of diabetes treatment. We gained valuable understanding of individual motivators that encourage PA. Important motivators were fear of complications, feeling of self-efficacy and enjoyment of PA. However, we also uncovered the potential negative impact of extreme awareness of self-efficacy, which can introduce pressure and feeling of guilt (self-efficacy paradox). Furthermore, our study identified key barriers to engaging in PA, including limiting comorbidities, lack of companionship, time constraints, and personal factors (e.g., perceived “laziness”). We also identified facilitators that GPs could leverage to help address these barriers, such as group-based support, the use of fitness trackers, and individualised guidance.

### Comparison with existing literature

#### Motivators and barriers

The motivators and barriers to engaging in PA among individuals living with T2DM are complex and multifaceted. In our study, key motivators included fear of complications, perceived self-efficacy, enjoyment of PA, and goals (concrete PA goals, medical and physical goals). At the same time, participants described limiting comorbidities, lack of companionship, time constraints, and discouraging emotions or low motivation as major barriers. These results echo the findings from broader literature on chronic disease management. A systematic review on barriers and facilitators to diet, PA and lifestyle behaviour intervention adherence across various health conditions revealed similar motivators, such as health concerns, physical change, social support and, self-regulation [21]. Specific research focusing on diabetes has identified common barriers, including time constraints, personal inertia, absence of exercise partners, and physical limitations, as well as motivators like self-efficacy and social support [22]. Additionally Blicher-Hansen et al. emphasised the roles of goal setting and intrinsic enjoyment of PA in facilitating long-term adherence among individuals living with T2DM [9].

The views on the role of rewards as motivators for PA were varied among our interview partners. While some found rewards motivating, others rejected them entirely. Although rewards are not highlighted as a primary motivator in broader discussions, the Compensatory Carry-Over Action Model (CCAM) by Lippke offers relevant insights. This model illustrates how different behaviours can interrelate within the context of achieving higher-level goals. According to this framework, rewards, such as food treats for exercising, could potentially motivate subsequent PA, or conversely, regular PA could encourage healthier eating habits [23].

#### Anxiety as motivator

Health concerns can serve as motivating factors for PA, particularly in managing chronic conditions like T2DM. Our study emphasises that beyond health concerns a strong feeling of anxiety about diabetes complications serves as a potent motivator, a finding that resonates with the observations by Blicher-Hansen et al. They noted that the negative emotional response triggered by understanding the chronic and progressive nature of T2D can spur of individuals living with T2DM into action [9]. However, anxiety as a motivator for PA in diabetes is rarely discussed, therefore the strong wording used by our interview partners was surprising for us. In broader research, anxiety is discussed as motivator for health behaviour. According to the Health Belief Model, perceived susceptibility influences the likelihood action [20]. For example, Wang et al. found that health anxiety positively affected exercise intention among healthy Chinese students and Zhang et al. reported that risk perception correlated with various health-protective behaviours beyond PA [24, 25].

Research shows that there is a heightened risk of anxiety disorders among individuals living with diabetes, suggesting a bidirectional relationship [26, 27]. Therefore, we think our findings on anxiety as motivator for PA are highly important.

#### Self-efficacy and psychological burdens

Feeling of self-efficacy can promote PA but can also introduce psychological burdens (self-efficacy paradox). Our study revealed instances where self-efficacy led to excessive self-monitoring or increased personal responsibility among individuals living with diabetes.

One of our interview partners described excessive self-monitoring. While research highlights the benefits of fitness trackers in PA, there are also reports of negative effects such as anxiety or frustration when users are unable to use their devices [28]. The growing market for such devices, e.g. the trending use of continuous glucose monitoring on social media—even among those without diabetes—has been discussed as potentially contributing to disordered eating in some cases. For instance, Wallace et al. demonstrated in a qualitative study that flash glucose monitoring can influence eating habits both positively and negatively in individuals living with type 1 diabetes, including ignored hunger urges and feelings of guilt [29]. In addition, Ogonesova et al., in their review, reported risks such as maladaptive dietary changes, anxiety, compulsive behaviours, or disordered eating patterns [30]. These and our findings on excessive self-monitoring suggest that while self-monitoring technologies can support diabetes management, they may also carry psychological risks that warrant further exploration.

Furthermore, our findings indicate a feeling of increased personal responsibility. One interview partner was directly being accused to be responsible for not passing diabetes to her daughter during pregnancy. These findings align with recent studies indicating that individuals living with T2DM often face criticism and blame related to their condition, leading to internalised frustration and negative self-perceptions [31, 32]. Another example is the heightened vigilance about disease prevention seen in our participant, Heidi, who feared transmitting diabetes to her children, may have inadvertently contributed to her daughter developing an eating disorder.

These cases illustrate the complex interplay between self-efficacy and the psychological impacts of diabetes management. The Health Belief Model by Rosenstock also identifies self-efficacy and its connection to perceived susceptibility as a key factor influencing the likelihood of engaging in health-related behaviour, however the model does not address the potential negative influence of high self-efficacy [20]. Moreover, our findings aligns with the Extended Parallel Process Model, which state that fear appeals can lead to health-protective behaviours when individuals perceive both a significant threat and a high level of efficacy to act on it [33].

#### Role of GP

The role of GPs as the primary contact for individuals living with T2DM is crucial. Our findings underscore the multifaced role of GPs, highlighting their potential to actively support PA. Interview participants detailed several practical strategies GPs could employ including personalised PA recommendations, goal-setting collaborations, assistance in finding PA partners, organising PA groups, and distributing informational flyers.

Furthermore, our findings reveal a diverse array of expectations and preferences regarding the doctor-patient relationship. In general, former diabetes research shows that patient-centred care has the potential to improve surrogate parameters like HbA1C [34]. However, our interviewees expressed a dual appreciation for both partnership-based and authoritative approaches in their interactions with healthcare providers. This dichotomy underscores the need for a balanced approach in counselling, integrating both support and guidance. Similar findings are observed in other studies, where rigid or misaligned doctor-patient dynamics can hinder the effectiveness of PA counselling, potentially leading to poor adherence and patient dissatisfaction [14].

### Implication for research and clinical practice

Our findings have various implications for research and clinical practice.

Research should further delve into the emotional and psychological impacts of diabetes management, such as anxiety and guilt, to better understand how these factors influence the adherence of individuals living with T2DM to prescribed PA regimes, overall management plans and quality of life. Another crucial area identified is the improvement of PA counselling in primary care, taking into account the specific motivators and barriers highlighted in our study.

In clinical practice, the implications of our findings suggest that clinicians need to tailor their communication and intervention strategies to meet the unique needs of each individual living with T2DM, taking into account their personal preferences. Elements of PA support could also be delivered through team-based care. For example, practice nurses and physiotherapists could provide individualised advice and support patients in managing hindering comorbidities. For GPs it is also important to find a balance in their roles, combining authority with supportiveness to motivate individuals living with T2DM effectively while providing empathetic care. Furthermore, integrating structured goal-setting into regular consultations can help individuals living with T2DM set and achieve realistic PA targets, thereby improving their motivation and adherence. Finally, it is important for GPs to be mindful of potential psychological comorbidities, as feelings of anxiety, frustration, and perceived stigma were prominently expressed by our interview participants. Recognising and addressing these psychological factors can also play a crucial role in effectively promoting PA.

### Strengths and weaknesses of the study

This study’s major strength lies in its qualitative approach, which provides deep insights into the personal perceptions, ideas, and wishes of individuals living with T2DM. Additionally, the diverse sample, including participants from various backgrounds, with different ages and different levels of PA, enhances transferability of the findings to the broader population of individuals living with T2DM in German primary care.

Despite these strengths, the study has limitations that should be considered. First, the recruitment via self-help groups and the fact that the participants knew that the interview was on PA might have introduced a selection bias. E.g. individuals with more severe complications or a negative view on PA might have different experiences and barriers related to PA. Additionally, all interviews were conducted via telephone due to COVID-19 restrictions, which might have limited the depth of interactions compared to face-to-face interviews. Moreover, some interviews were relatively short (< 45 min), which may have further limited the depth of exploration. This may partly reflect the telephone format and/or interviewer experience. Furthermore, while the diversity of the sample can be considered a strength, it may also have made it more challenging to fully saturate all participant subgroups, which could limit the transferability of the findings. Finally, while the study provides valuable insights into how individuals living with T2DM perceive the role of GPs in facilitating PA, it does not include perspectives from the GPs themselves, which could provide a more rounded view of the potential for primary care interventions.

## Conclusion

Our study highlights the crucial role of GPs in promoting PA as a fundamental aspect of managing T2DM. We identified numerous challenges and barriers in PA counselling, spanning psychological difficulties such as anxiety and practical issues like time constraints. Additionally, we identified motivators and facilitators that could enhance PA. These findings aim to improve health outcomes and quality of life for individuals living with T2DM through better support in primary care settings.

## Supplementary Information

Supplementary Material 1.

Supplementary Material 2.

## Data Availability

The raw interview data are not publicly available, as participants did not consent to data sharing.

## Abbreviations

GP: general practitioners
PA: physical activity
T2DM: Type 2 Diabetes Mellitus
