# Supplementary material for: Physical activity and diabetes in german primary care: a qualitative interview study with individuals living with diabetes
Source: BMC Prim Care. 2026 Apr 29;27:169. doi: 10.1186/s12875-026-03344-z (PMC13130814; doi:10.1186/s12875-026-03344-z)
Supplement: Supplementary file 2 — Supplementary Material 2. [file 12875_2026_3344_MOESM2_ESM.docx]

# Supplement 1 interview guide

The original guide was German. For publication it was translated in English

### **Main Questions (with follow-up prompts)**

#### **1. Own Physical Activity**

- **How satisfied are you with your current physical activity in everyday life?**
- Please list the kinds of daily/regular physical activities you do or any activity groups you attend.
- What are your goals for being physically active? (e.g., blood sugar, fitness, etc.)
- Would you like to be more active? If yes, why?

#### **2. Type 2 Diabetes**

- **What impact does diabetes have on your physical activity?**
- How physically active were you before your diagnosis?
- Do you feel you can positively influence the course of your illness? In what way?
- Have you participated in any diabetes education programs or other relevant offerings?

#### **3. GP Practice Setting**

- **Do you think the GP practice is a suitable setting for promoting physical activity? Why or why not**?
- Should the GP practice play a coordinating role?

#### **4. Conversations with the GP to Promote Activity**

- How often should such conversations occur?
- (Has such a conversation ever taken place?)
- How important is your relationship with your GP in lifestyle counseling?
- (How would you describe your relationship with your GP?)
- Should decisions be made jointly or by the GP alone?
- Where could your GP start to motivate you to be more active? What is your "tender spot"? How could your GP "tease out" more motivation?
- What advice from your GP has already helped you become more active?

#### **5. Information**

- What kind of materials would be helpful? (flyers, brochures, magazines, websites, videos)
- What content should be included?
- How should the information be delivered? (in waiting room, by mail, handed over personally, etc.)

#### **6. Exercise Classes via the GP Practice (Theoretical Offer)**

- What would you like to learn about?
- How often should it take place?
- Led by GP, medical assistant, or external instructor?
- Would it be better if participation was voluntary or mandatory?
- Reports from other people with diabetes – discouraging or motivating?

#### **7. Physical Activity Programs via the GP Practice**

- Which types of activities would you prefer? (e.g., walking group)
- Where should it take place? (How far would you travel?)
- How should such an offer ideally look?
- Led by GP, medical assistant, or external instructor?
- Group or individual setting? Why? (competition or distraction?)
- What about the offer would motivate you?
- How often should it take place?
- Regular, continuous offer with flexible participation or fixed group? (If fixed group, is the sense of obligation helpful or not?)
- Should participation be mandatory?
- (Other therapy options?)
- (Rehabilitation sports – how often/where?)

#### **8. Financial Support**

**Memberships**

- Under what circumstances would you accept and use financial support?
- Should it be fully funded or partially subsidized?
- What would be most helpful? (gym membership, swim passes, yoga, group exercise, etc.)

**Bonus Program for Activity**

- Financial incentives for high scores. What kind? (e.g., reduced insurance premiums)
- Would that motivate you to be more active?

**Provision of Equipment**

- Would it help if equipment were provided (fitness tracker, pedometer, hiking poles, sports shoes, etc.)?

**Apps or Online Offers**

### **Closing**

Please take a moment: Do you have any additional ideas on how your GP could support you?

How likely do you think it is that you’ll succeed in becoming more active?
